# One year of liraglutide treatment offers sustained and more effective glycaemic control and weight reduction compared with sitagliptin, both in combination with metformin, in patients with type 2 diabetes: a randomised, parallel-group, open-label trial

**DOI:** 10.1111/j.1742-1241.2011.02656.x

**Published:** 2011-04

**Authors:** R Pratley, M Nauck, T Bailey, E Montanya, R Cuddihy, S Filetti, A Garber, A B Thomsen, H Hartvig, M Davies, for the 1860-LIRA-DPP-4 Study Group

**Affiliations:** 1 Diabetes and Metabolism Translational Medicine Unit, University of Vermont College of MedicineBurlington, VT, USA; 2 Diabetes Center, Diabeteszentrum Bad Lauterberg, Bad Lauterberg im HarzGermany; 3 Director, AMCR InstituteEscondido, CA, USA; 4 Endocrine Unit, IDIBELL-Hospital Universitari BellvitgeBarcelona, Spain; 5 Medical Director, International Diabetes CenterMinneapolis, MN, USA; 6 Department of Clinical Sciences, Sapienza University of RomeRome, Italy; 7 Departments of Medicine, Biochemistry, and Molecular and Cellular Biology, Baylor College of MedicineHouston, TX, USA; 8 Medical & Science Department, Novo Nordisk A/SSoeborg, Denmark; 9 Biostatistical Department, Novo Nordisk A/SSoeborg, Denmark; 10 Department of Cardiovascular Sciences, University of LeicesterLeicester, UK

## Abstract

**Aim:**

The aim of this study was to compare the efficacy and safety of once-daily human glucagon-like peptide-1 analogue liraglutide with dipeptidyl peptidase-4 inhibitor sitagliptin, each added to metformin, over 52 weeks in individuals with type 2 diabetes.

**Methods:**

In an open-label, parallel-group trial, metformin-treated participants were randomised to liraglutide 1.2 mg/day (*n*= 225), liraglutide 1.8 mg/day (*n*= 221) or sitagliptin 100 mg/day (*n*= 219) for 26 weeks (main phase). Participants continued the same treatment in a 26-week extension.

**Results:**

Liraglutide (1.2 or 1.8 mg) was superior to sitagliptin for reducing HbA_1c_ from baseline (8.4–8.5%) to 52 weeks: −1.29% and −1.51% vs. −0.88% respectively. Estimated mean treatment differences between liraglutide and sitagliptin were as follows: −0.40% (95% confidence interval −0.59 to −0.22) for 1.2 mg and −0.63% (−0.81 to −0.44) for 1.8 mg (both p < 0.0001). Weight loss was greater with liraglutide 1.2 mg (−2.78 kg) and 1.8 mg (−3.68 kg) than sitagliptin (−1.16 kg) (both p < 0.0001). Diabetes Treatment Satisfaction Questionnaire scores increased significantly more with liraglutide 1.8 mg than with sitagliptin (p = 0.03). Proportions of participants reporting adverse events were generally comparable; minor hypoglycaemia was 8.1%, 8.3% and 6.4% for liraglutide 1.2 mg, 1.8 mg and sitagliptin respectively. Gastrointestinal side effects, mainly nausea, initially occurred more frequently with liraglutide, but declined after several weeks.

**Conclusion:**

Liraglutide provides greater sustained glycaemic control and body weight reduction over 52 weeks. Treatment satisfaction was significantly greater with 1.8 mg liraglutide, similar to 26-week results. The safety profiles of liraglutide and sitagliptin are consistent with previous reports.

**What's known**

Results of independent trials and several 26-week head-to-head trials suggest that GLP-1 receptor agonists produce greater glycaemic and weight reductions compared with DPP-4 inhibitors. Our 26-week trial showed that the human once-daily GLP-1 analogue liraglutide effected greater glycaemic control and weight loss than the DPP-4 inhibitor sitagliptin.

**What's new**

Longer-term sustainability of the 26-week efficacy and safety results with liraglutide and sitagliptin, as well as the maintenance of the greater comparative efficacy with liraglutide, was not known. This report shows that 26-week improvements were sustained after 52 weeks of treatment, with liraglutide producing significantly greater glycaemic and weight reductions than sitagliptin.

## Introduction

Metformin is now standard first-line treatment (in addition to lifestyle modifications) for type 2 diabetes (T2D) (1). The progressive nature of T2D, including declining beta-cell function, usually necessitates addition of other antihyperglycaemic agents to metformin, as blood glucose levels rise. However, current guidelines vary with respect to second-line therapy (1,2). A meta-analysis of currently available non-insulin antihyperglycaemic agents added to metformin revealed that, while reductions in glycosylated haemoglobin (HbA_1c_) were similar across several drug classes (including sulphonylureas, thiazolinediones and alpha-glucosidase inhibitors; reduction range: 0.64–0.97%), treatment side effects (such as weight gain and/or hypoglycaemia) varied considerably (3). Therefore, head-to-head studies of glucose-lowering agents are needed to compare overall clinical efficacy and safety when added to metformin.

Treatment intensification with incretin-based therapies is appealing given that they provide good glycaemic control with a low risk of hypoglycaemia, because of the glucose-dependent stimulation of insulin secretion and inhibition of glucagon release, and do not produce weight gain (3–6). Glucagon-like peptide-1 (GLP-1) receptor agonists and dipeptidyl peptidase-4 (DPP-4) inhibitors are two distinct classes of incretin-based therapies. While 26-week, head-to-head studies suggest that GLP-1 receptor agonists have greater glycaemic and weight reduction efficacy than DPP-4 inhibitors (7–9), longer-term results have not been reported.

In a 26-week, head-to-head trial of the once-daily human GLP-1 analogue liraglutide and the DPP-4 inhibitor sitagliptin, both in combination with metformin, liraglutide (1.2 or 1.8 mg/day) was significantly more effective than sitagliptin (100 mg/day) for reducing HbA_1c_ (−1.24% and −1.50% vs. −0.90% respectively), fasting plasma glucose (FPG) (−1.87 mmol/l [−33.66 mg/dl] and −2.14 mmol/l [−38.52 mg/dl] vs. −0.83 mmol/l [−14.94 mg/dl], respectively) and body weight (−2.86 and −3.38 kg vs. −0.96 kg respectively) (8). Incidence of minor hypoglycaemia was low (around 5%) and comparable across treatment groups. Nausea incidence was greater with liraglutide than with sitagliptin during therapy initiation, but generally declined after several weeks of treatment.

Trial participants could continue treatment in a 26-week extension phase designed to evaluate the sustainability of efficacy and safety effects of liraglutide and sitagliptin. This report shows that 26-week improvements were sustained after 52 weeks of treatment, with liraglutide producing greater glycaemic and weight reductions than sitagliptin.

## Methods

### Study design

Details of study design and participant inclusion/exclusion criteria have been reported previously (8). Briefly, in a multinational, randomised, parallel-group, open-label, active-comparator trial, participants with T2D previously treated with metformin monotherapy (≥ 1500 mg/day) for a minimum of 3 months, but with suboptimal glycaemic control (HbA_1c_ 7.5–10%), were randomised (1 : 1 : 1) to treatment with either liraglutide 1.2 or 1.8 mg/day (subcutaneous injection) or sitagliptin 100 mg/day (orally) while continuing on existing metformin therapy.

After completing the 26-week main phase, participants choosing to enrol in the extension provided written informed consent and continued for another 26 weeks in their originally assigned treatment groups. The protocol, including the extension, was institutional review board-approved, followed Good Clinical Practice guidelines and conformed to the Declaration of Helsinki. The 52-week trial was initiated on 16 June 2008 and completed on 10 December 2009.

Additional withdrawal criteria during the extension were: elevated FPG > 11.1 mmol/l (200 mg/dl) with no treatable intercurrent cause or acute pancreatitis (defined as a minimum two out of three of the following: characteristic abdominal pain, amylase and/or lipase > 3 × upper normal range or characteristic findings on computed tomography/magnetic resonance imaging).

### Outcomes

Efficacy outcomes assessed at 52 weeks included change in HbA_1c_, FPG, body weight, proportion of participants achieving HbA_1c_ < 7% or ≤ 6.5%, proportion of participants reaching the composite end-point of HbA_1c_ < 7.0% with no weight gain and no confirmed major (participant unable to treat him/herself) or minor (plasma glucose < 3.1 mmol/l [56 mg/dl]) hypoglycaemia. Other measures included fasting C-peptide, fasting pro-insulin : insulin ratio, and homeostasis model assessment analyses of beta-cell function (HOMA-B) and insulin resistance (HOMA-IR). Change in Diabetes Treatment Satisfaction Questionnaire (DTSQ) scores from baseline was not assessed in participants from Slovakia, Serbia or Slovenia (118/665 [17.7%]) because of the lack of validated questionnaires in their native languages.

Safety and tolerability assessments at 52 weeks included incidence of adverse events (AEs) and hypoglycaemia, as well as various clinical and laboratory variables. AEs of special interest included nausea, thyroid AEs and pancreatitis.

### Statistical analysis

Methods for statistical analyses were similar to those reported for the first 26 weeks (8). Glycaemic efficacy, as measured by change in HbA_1c_ from baseline to week 52 of liraglutide vs. sitagliptin treatment, was assessed by a non-inferiority comparison with a margin of 0.4%, followed by a superiority comparison. Both tests used two-sided hypotheses, with a p-value of < 0.05 considered significant. Analysis of covariance, with treatment and country as fixed effects and baseline measure as a covariate, was used for continuous efficacy end-points. Logistic regression was used to analyse categorical variables, including the participant proportions achieving HbA_1c_ targets and composite end-point (HbA_1c_ < 7.0% with no weight gain and no confirmed major or minor hypoglycaemia), with treatment and country as fixed effects, and baseline HbA_1c_ (and body weight for composite) as covariates. Efficacy assessments were performed on the full analysis set: all randomised participants exposed to at least one dose of the drug. Missing data were imputed using the last observation carried forward (LOCF) method.

The safety analysis set included all participants exposed to at least one dose of the drug they were randomised to. Serum calcitonin values were analysed using a repeated measures model, with time, gender, treatment and treatment-by-time interaction as fixed effects and participant as a random effect. Hypoglycaemia was analysed using a general linear model with treatment as a fixed effect. For each week of the extension, the proportions of participants experiencing nausea were analysed using Fisher's exact test. Only summary statistics are reported for other safety parameters. Data are reported as least square means with 95% confidence interval (CI), unless otherwise noted. The significance level is p < 0.05.

## Results

### Participant disposition and baseline characteristics

**Figure 1:**
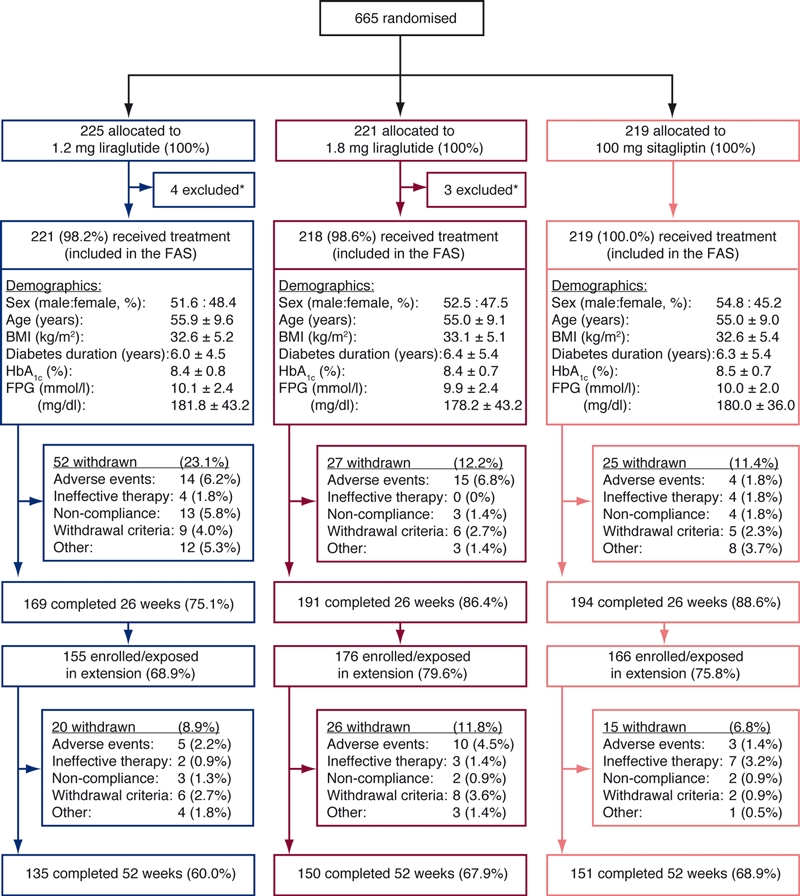
Trial flow chart with participant demographics at baseline. Demographic data are mean ± SD, unless otherwise noted. *Participants were withdrawn if they fulfilled withdrawal criteria, decided that they no longer wanted to participate, or did not attend any visit after randomisation. BMI, body mass index; FAS, full analysis set; FPG, fasting plasma glucose

After screening, 665 participants were randomised into three treatment arms: liraglutide 1.2 mg, 1.8 mg and sitagliptin (Figure 1). As previously reported, the groups were well matched for baseline characteristics (8). Of participants completing 26 weeks, 497/554 (90%) entered into the extension, with 436/497 (88%) completing 52 weeks. A lower proportion of randomised participants withdrew from the extension compared with the main phase, and withdrawal because of AEs was also lower in the extension. Patient withdrawal because of AEs in the main phase was higher for both liraglutide groups than for sitagliptin, whereas only the liraglutide 1.8 mg group had a slightly higher AE withdrawal rate in the extension.

### Efficacy outcomes

**Figure 2:**
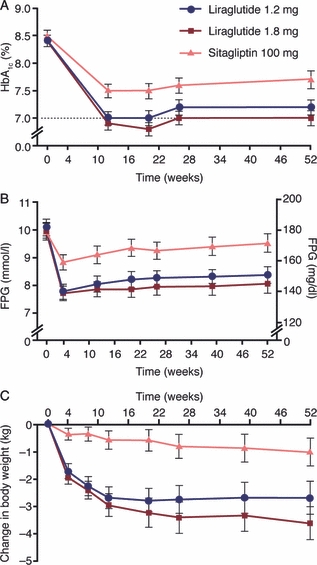
Effect of 1.2 mg liraglutide, 1.8 mg liraglutide or 100 mg sitagliptin on glycaemic control and body weight from baseline to 52 weeks. (A) Mean HbA_1c_ values. (B) Mean fasting plasma glucose (FPG) values. (C) Mean change in body weight. Error bars are 1.96 × SE, corresponding to the 95% CI

Mean HbA_1c_ decreased more substantially with either dose of liraglutide compared with sitagliptin during the first 12 weeks, and these reductions were generally maintained up to week 52 (Figure 2A). Mean reductions in HbA_1c_ from baseline to week 52 with liraglutide 1.2 mg (−1.29% [95% CI: −1.43 to −1.15]) and 1.8 mg (−1.51% [−1.65 to −1.37]) were significantly greater compared with sitagliptin (−0.88% [−1.02 to −0.74]). Estimated mean treatment differences were −0.40% (95% CI −0.59 to −0.22) for liraglutide 1.2 mg vs. sitagliptin and −0.63% (−0.81 to −0.44) for liraglutide 1.8 mg vs. sitagliptin (p < 0.0001 for both doses.)

As with HbA_1c_, liraglutide was more effective for reducing FPG compared with sitagliptin (Figure 2B). FPG declined rapidly from baseline during weeks 0–4 in all treatment groups and the reductions were generally sustained up to 52 weeks. FPG reductions from baseline at week 52 were −1.71 mmol/l (95% CI −2.04 to −1.38) (−30.78 mg/dl [−36.78 to −24.78]) for 1.2 mg liraglutide and −2.04 mmol/l (−2.37 to −1.71) (−36.72 mg/dl [−42.72 to −30.72]) for 1.8 mg liraglutide vs. −0.59 mmol/l (−0.92 to −0.26) (−10.62 mg/dl [−16.62 to −4.62]) for sitagliptin. Estimated mean treatment differences between liraglutide and sitagliptin were −1.13 mmol/l (95% CI −1.57 to −0.68) (−20.34 mg/dl [−28.26 to −12.24]) for 1.2 mg and −1.45 mmol/l (−1.89 to −1.01) (−26.1 mg/dl [−34.02 to −18.18]) for 1.8 mg (p < 0.0001 vs. sitagliptin for both doses).

Weight loss was considerably greater with liraglutide compared with sitagliptin (Figure 2C). Most weight loss occurred during the first 26 weeks and was sustained in the extension in all treatment groups (Figure 2C). At week 52, weight loss with liraglutide 1.2 mg was −2.78 kg (95% CI −3.39 to −2.17) compared with −3.68 kg (−4.29 to −3.07) for 1.8 mg and −1.16 kg (−1.77 to −0.55) for sitagliptin. Estimated mean treatment differences were −1.62 kg (95% CI −2.43 to −0.82) for liraglutide 1.2 mg and −2.53 kg (−3.33 to −1.72) for liraglutide 1.8 mg vs. sitagliptin (p < 0.0001 for both doses). Weight loss with liraglutide 1.8 mg was significantly greater than that with liraglutide 1.2 mg (p = 0.03). The 26-week-reductions in waist circumference were generally maintained at week 52 in all groups and were significantly larger with liraglutide (both doses) than sitagliptin (Table 1).

**Table 1:** Changes in secondary end-points from baseline to week 52

	**Mean change from baseline [95% CI]**			**Estimated treatment difference; p-value**	
		
	**Liraglutide 1.2 mg**	**Liraglutide 1.8 mg**	**Sitagliptin 100 mg**	**Liraglutide 1.2 mg–sitagliptin**	**Liraglutide 1.8 mg–sitagliptin**
**Beta-cell function and insulin resistance**					
Fasting insulin (pmol/l)	−0.60 (−10.11 to 8.91)	1.63 (−7.74 to 11.00)	−2.27 (−11.76 to 7.22)	1.67 (−11.2 to 14.51); 0.80	3.90 (−8.76 to 16.57); 0.55
Fasting C-peptide (nmol/l)	0.05 (−0.01 to 0.11)	0.09 (0.03 to 0.15)	0.01 (−0.05 to 0.07)	0.04 (−0.05 to 0.12); 0.38	0.08 (0.00 to 0.16); 0·06
Fasting pro-insulin:insulin ratio	−0.07 (−0.11 to −0.03)	−0.09 (−0.13 to −0.05)	−0.01 (−0.05 to 0.03)	−0.06 (−0.11 to −0.02); 0.005	−0.08 (−0.13 to −0.04); < 0.001
HOMA-B (%)	22.58 (16.09 to 29.07)	25.76 (19.39 to 32.13)	3.98 (−2.45 to 10.45)	18.60 (9.82 to 27.38); < 0.001	21.77 (13.12 to 30.42); < 0.001
HOMA-IR (%)	−1.27 (−1.96 to −0.58)	−1.36 (−2.05 to −0.67)	−0.41 (−1.1 to 0.28)	−0.85 (−1.79 to 0.08); 0.07	−0.94 (−1.86 to −0.02); 0.04
**Blood pressure**					
Systolic blood pressure (mmHg)	−0.37 (−2.19 to 1.45)	−2.55 (−4.37 to −0.72)	−1.03 (−2.85 to 0.79)	0.66 (−1.79 to 3.10); 0.60	−1.53 (−3.97 to 0.92); 0.22
Diastolic blood pressure (mmHg)	–0.53 (−1.65 to 0.59)	−0.87 (−1.99 to 0.25)	−1.47 (−2.59 to −0.35)	0.94 (−0.57 to 2.45); 0.22	0.60 (−0.90 to 2.11); 0.43
Heart rate (beats/min)	1.72 (0.54 to 2.90)	3.09 (1.91 to 4.27)	0.09 (−1.09 to 1.27)	1.63 (0.03 to 3.22); 0.05	3.00 (1.40 to 4.59); < 0.001
**Lipid profiles**					
Total cholesterol (mmol/l)	−0.01 (−0.13 to 0.11)	−0.09 (−0.21 to 0.03)	0.03 (−0.15 to 0.09)	−0.04 (−0.20 to 0.12); 0.61	−0.12 (−0.28 to 0.03); 0.12
LDL cholesterol (mmol/l)	0.09 (−0.01 to 0.19)	0.09 (−0.01 to 0.19)	0.17 (0.07 to 0.27)	−0.08 (−0.20 to 0.05); 0.25	−0.08 (−0.21 to 0.05); 0.23
VLDL cholesterol (mmol/l)	−0.11 (−0.19 to −0.03)	−0.19 (−0.27 to −0.11)	−0.15 (−0.23 to −0.07)	0.04 (−0.06 to 0.13); 0.47	−0.04 (−0.14 to 0.06); 0.43
HDL cholesterol (mmol/l)	0.01 (−0.01 to 0.03)	0.02 (0.00 to 0.04)	0.01 (−0.01 to 0.03)	0.00 (−0.03 to 0.04); 0.92	0.01 (−0.02 to 0.05); 0.53
Triglycerides (mmol/l)	−0.10 (−0.30 to 0.10)	−0.32 (−0.52 to −0.12)	−0.23 (−0.43 to −0.03)	0.12 (−0.13 to 0.38); 0.34	−0.09 (−0.34 to 0.16); 0.49
Free fatty acids (mmol/l)	−0.07 (−0.11 to −0.03)	−0.10 (−0.14 to −0.06)	−0.06 (−0.10 to −0.02)	−0.01 (−0.06 to 0.04); 0.67	−0.05 (−0.09 to 0.00); 0.05
Apolipoprotein B (g/l)	−0.03 (−0.05 to −0.01)	–0.03 (−0.05 to −0.01)	–0.03 (−0.05 to −0.01)	0.00 (−0.03 to 0.04); 0.83	−0.00 (−0.04 to 0.03); 0.93
**Physical measure(s)**					
Waist circumference (cm)	−2.36 (−3.10 to −1.62)	−3.02 (−3.76 to −2.28)	−1.23 (−1.97 to −0.49)	−1.13 (−2.12 to −0.13); 0·03	−1.79 (−2.78 to −0.79); < 0.001

Data are least square means (95% CI); p-values (for treatment differences). CI, confidence interval; HDL, high-density lipoprotein; HOMA-B, homeostasis model assessment of beta-cell function; HOMA IR, HOMA insulin resistance; LDL, low-density lipoprotein; VLDL, very low-density lipoprotein.

As with the main study results (8), postprandial plasma glucose data were highly variable and difficult to interpret, and are excluded from this report. As this was a multinational study, data variability may have resulted from the varying meal content, time of meals and timing of postprandial glucose measurements across different countries.

**Figure 3:**
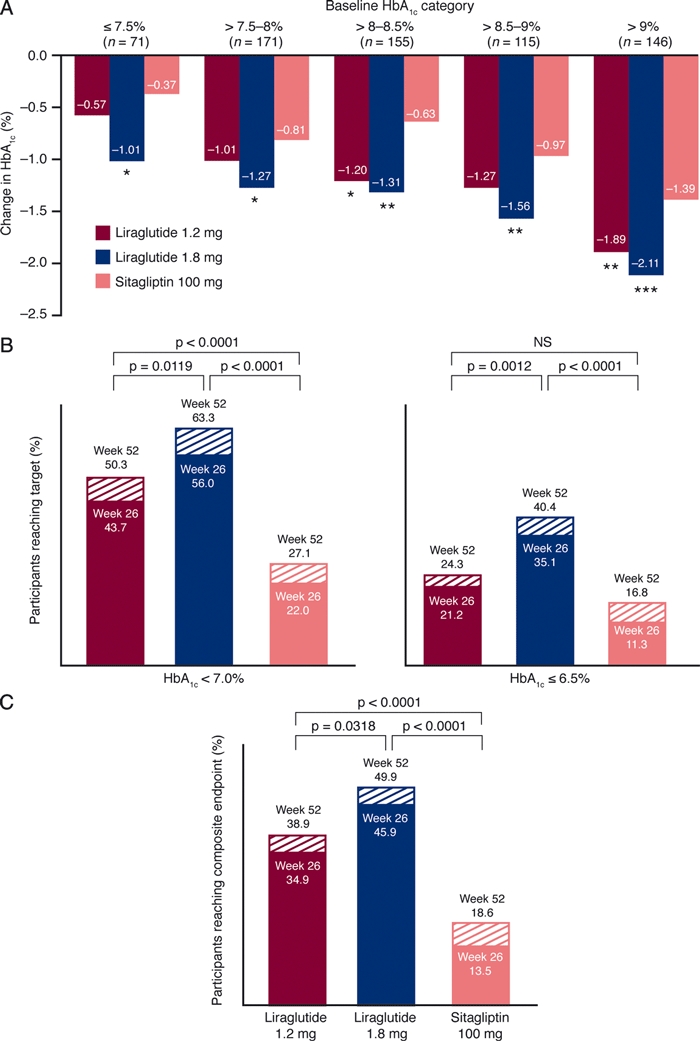
Mean reductions in HbA_1c_ by baseline category and proportions of participants reaching target end-points with 1.2 mg liraglutide, 1.8 mg liraglutide and 100 mg sitagliptin from weeks 0–52. (A) Mean reductions in HbA_1c_ from baseline to week 52 by baseline HbA_1c_ category. (B) Percentage of participants achieving target HbA_1c_ < 7% (ADA) or ≤ 6.5% (AACE). (C) Percentage of participants reaching the composite end-point of HbA_1c_ < 7.0%, with no weight gain and no confirmed major or minor hypoglycaemia. In (B) and (C), solid bar portions represent percentages from weeks 0–26, while shaded portions represent percentages from weeks 27–52. *p < 0.05 vs. sitagliptin; **p ≤ 0.01 vs. sitagliptin; ***p < 0.001 vs. sitagliptin. p-values are derived from a logistic regression model with treatment and country as fixed effects and baseline HbA_1c_ and body weight (for composite) as covariate(s). AACE, American Association of Clinical Endocrinologists; ADA, American Diabetes Association

Overall, the magnitude of HbA_1c_ reduction from baseline increased with the higher baseline HbA_1c_ categories in all groups (Figure 3A). After 52 weeks, mean reductions in HbA_1c_ were significantly greater with liraglutide 1.8 mg than with sitagliptin across all baseline HbA_1c_ categories. The reductions were significantly larger with liraglutide 1.2 mg than with sitagliptin for two baseline HbA_1c_ categories: > 8% to ≤ 8.5% and > 9%.

Proportions of participants achieving target HbA_1c_ < 7% (American Diabetes Association [ADA] target) or ≤ 6.5% (American Association of Clinical Endocrinologists [AACE] target) increased during the extension in all treatment groups (Figure 3B). Overall, liraglutide (both doses) was significantly more effective than sitagliptin in allowing patients to reach target HbA_1c_ after 52 weeks.

The estimated proportion of participants reaching the composite end-point of HbA_1c_ < 7.0%, with no weight gain and no confirmed major or minor hypoglycaemia, increased during the extension in all treatment groups (Figure 3C). After 52 weeks, a significantly greater percentage of participants achieved the composite end-point with liraglutide (both groups) than with sitagliptin, with an odds ratio (OR) vs. sitagliptin of 2.80 (95% CI 1.74 to 4.48) and 4.37 (2.74 to 6.98) for 1.2 and 1.8 mg liraglutide respectively (both doses p < 0.0001). Liraglutide 1.8 mg was more effective than liraglutide 1.2 mg (OR: 1.56 [1.04 to 2.35], p = 0.03).

Overall, the improved status of several indicators of beta-cell function (fasting C-peptide, fasting pro-insulin:insulin ratio and HOMA-B) at week 26 was maintained at week 52, with liraglutide effecting significantly greater improvements than sitagliptin (Table 1). The reduction in HOMA-IR became significantly greater with liraglutide 1.8 mg than sitagliptin during the extension. As observed at week 26, mean heart rate continued to be slightly but significantly elevated with liraglutide compared with sitagliptin at week 52 (Table 1).

The increase in DTSQ scores at week 26 was generally sustained at week 52 in all treatment groups. The improvement in overall treatment satisfaction, measured as the increase in DTSQ scores between weeks 0 and 52, was significantly higher with liraglutide 1.8 mg (baseline: 28.0) than with sitagliptin (baseline: 27.1): 4.3 (95% CI 3.3 to 5.3) vs. 3.0 (2.0 to 4.0) (p = 0.03). By contrast, the increase from baseline (27.8) with liraglutide 1.2 mg (3.3 [2.3 to 4.3]) was not statistically different from sitagliptin.

### Safety outcomes

The majority (≥ 97%) of treatment-emergent AEs in all groups over 52 weeks were mild or moderate. The proportion of participants reporting serious AEs was low and comparable between treatment groups (4.5%, 6.0% and 5.5% for liraglutide 1.2 mg, 1.8 mg and sitagliptin respectively) with no consistent pattern with respect to system organ class (Table 2).

**Table 2:** Participants with treatment-emergent adverse events during weeks 0–52

**Adverse events**	**Liraglutide 1.2 mg/day (*n* = 221)**	**Liraglutide 1.8 mg/day (*n* = 218)**	**Sitagliptin 100 mg/day (*n* = 219)**
Serious adverse events*	10 (4.5)	13 (6.0)	12 (5.5)
Deaths	0	1 (0.5)	2 (0.9)
**Severe adverse events**	12 (5.4)	15 (6.9)	13 (5.9)
Gastrointestinal disorders	4 (1.8)	5 (2.3)	4 (1·8)
Musculoskeletal and connective tissue disorders	3 (1.4)	1 (0.5)	1 (0.5)
Infections and infestations	3 (1.4)	3 (1.4)	3 (1.4)
Neoplasms (benign, malignant and unspecified)	1 (0.5)	3 (1.4)	1 (0.5)
Cardiac disorders	2 (0.9)	1 (0.5)	1 (0.5)
Investigations	0 (0.0)	1 (0.5)	0 (0.0)
Nervous system disorders	0 (0.0)	0 (0.0)	2 (0.9)
Renal and urinary disorders	0 (0.0)	0 (0.0)	1 (0.5)
**Adverse events (of any severity) reported by > 5% of participants**	158 (71.5)	167 (76.6)	139 (63.5)
Gastrointestinal disorders	80 (36.2)	94 (43.1)	52 (23.7)
Nausea	48 (21.7)	60 (27.5)	12 (5.5)
Vomiting	18 (8.1)	23 (10.6)	11 (5.0)
Diarrhoea	20 (9.0)	27 (12.4)	14 (6.4)
Constipation	10 (4.5)	13 (6.0)	8 (3.7)
Dyspepsia	8 (3.6)	15 (6.9)	5 (2.3)
Infections and infestations	74 (33.5)	77 (35.3)	75 (34.2)
Nasopharyngitis	27 (12.2)	32 (14.7)	31 (14.2)
Influenza	13 (5.9)	4 (1.8)	8 (3.7)
Nervous system disorders	40 (18.1)	48 (22.0)	44 (20.1)
Headache	21 (9.5)	29 (13.3)	27 (12.3)
Musculoskeletal and connective tissue disorders	39 (17.6)	45 (20.6)	45 (20.5)
General disorders and administration-site conditions	31 (14.0)	32 (14.7)	13 (5.9)
Metabolism and nutrition disorders	25 (11.3)	27 (12.4)	19 (8.7)
Decreased appetite	8 (3.6)	12 (5.5)	3 (1.4)
Investigations	21 (9.5)	27 (12.4)	16 (7.3)
Skin and subcutaneous tissue disorders	22 (10.0)	20 (9.2)	22 (10.0)
Respiratory, thoracic and mediastinal disorders	16 (7.2)	18 (8.3)	21 (9.6)
Injury, poisoning and procedural complications	20 (9.0)	20 (9.2)	21 (9.6)
Vascular disorders	16 (7.2)	15 (6.9)	10 (4.6)

Data are number (%) of participants. A participant could experience more than one adverse event.

* Liraglutide 1.2 mg group: acute myocardial infarction, myocardial infarction, epiglottic carcinoma, thyroid disorder, hypertensive crisis (relapsing), hypoesthesia, coxarthrosis deformans, worsening of coxarthrosis, haemorrhagic anaemia, haematochezia, infected sebaceous cyst; liraglutide 1.8 mg group: pancreatic carcinoma (fatal), renal adenoma, breast cancer, colon cancer, heart failure, sepsis, chest discomfort, subdural haematoma, subileus, pneumonia, diabetic retinopathy and papilloedema, mycotic mycetoma of left sphenoidal sinus and epistaxis, right hip arthroplasty, cholecystitis, peritonitis, worsening of cervicocranial syndrome; sitagliptin group: cardiac arrest (fatal), sudden cardiac death (fatal), renal carcinoma, colonic polyp, postmenopausal vaginal haemorrhage and leiomyoma, worsening morbus Osler, worsening of sleep apnoea; right meniscus rupture, anal abscess, hernia inguinalis (left side), acute cholecystitis, dengue fever.

Three deaths occurred during the 52-week period. Two deaths during the first 26 weeks, one in a participant with pancreatic carcinoma (liraglutide 1.8 mg) and one because of cardiac arrest (sitagliptin), were reported previously and considered unlikely to be related to the trial drugs (8). One sudden cardiac death during the extension occurred in a 66-year-old man randomised to sitagliptin and was judged as unlikely to be related to the trial drug by the investigator.

**Figure 4:**
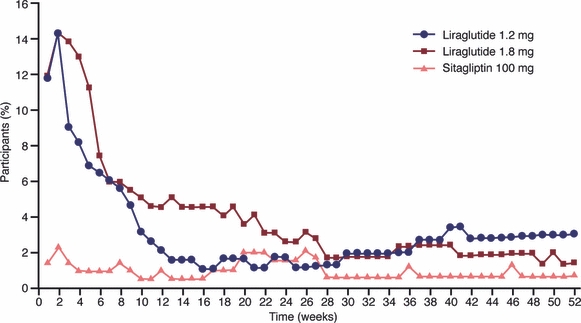
Weekly percentage of participants reporting nausea with 1.2 mg liraglutide, 1.8 mg liraglutide or 100 mg sitagliptin during weeks 0–52. Weekly proportions of participants experiencing nausea during the extension (weeks 27–52) did not differ significantly between liraglutide (1.2 or 1.8 mg) and sitagliptin treatment groups

Gastrointestinal disorders, as well as infections and infestations, were the most commonly reported mild-to-moderate AEs with liraglutide. The incidence of nausea, the most prevalent gastrointestinal AE with liraglutide, declined after the first 3 weeks of treatment and remained low during the extension (Figure 4). For each week of the extension, the proportions of participants experiencing nausea did not differ significantly between liraglutide (1.2 or 1.8 mg) and sitagliptin treatment groups.

One episode of major hypoglycaemia (blood glucose 3.6 mmol/l [64.8 mg/dl]) occurred during the first 26 weeks in a participant on liraglutide 1.2 mg (8). Third-party assistance was required, but no seizures or coma occurred. The participant recovered and the episode was categorised as possibly related to the trial product by the investigator. No major hypoglycaemic episodes occurred during the extension. Minor hypoglycaemia rates were low and comparable between treatment groups over 52 weeks, after excluding an outlier in the 1.8 mg liraglutide group with 21 minor events during the first 26 weeks and two events in the extension (leading to participant withdrawal from the trial). Adjusted minor hypoglycaemia rates were 0.143, 0.154 and 0.137 hypoglycaemic episodes per patient per year for liraglutide 1.2 mg, 1.8 mg and sitagliptin respectively.

One case of ‘non-acute’ pancreatitis was reported during the extension in a 54-year-old man, with a medical history of hepatitis and hyperlipidaemia, treated with liraglutide 1.8 mg for 227 days. Initially, the participant experienced abdominal pain, nausea, vomiting for 1 day and black stools for 3 days. The participant was instructed to stop aspirin and initiate omeprazole treatment. Upon later hospitalisation for a different condition, laboratory tests showed slightly increased levels of amylase (2.6 μkat/l, normal range: 0–1.67 μkat/l) and lipase (1.44 μkat/l, normal range: 0–1 μkat/l). The investigator decided to withdraw the participant, although the specific withdrawal criteria for acute pancreatitis were not met. The event was rated as mild and possibly related to the trial drug by the investigator.

Mean changes in serum calcitonin, an indicator of C-cell hyperplasia, were small and there were no statistically significant differences between treatment groups. Mean calcitonin levels remained below the upper normal limit for both genders from baseline to 52 weeks. The proportions of subjects reporting thyroid-related treatment-emergent AEs were comparable across treatment groups (5.0%, 5.5% and 4.6% for liraglutide 1.2 mg, 1.8 mg and sitagliptin respectively) (Table S1). No cases of thyroid malignancy were found during the trial period (Table S2).

## Discussion

Liraglutide produced sustained and greater reductions in HbA_1c_, FPG and body weight compared with sitagliptin after 52 weeks of treatment, similar to results previously reported after week 26 (8). Furthermore, liraglutide improved glycaemic control to a greater extent than sitagliptin irrespective of baseline HbA_1c_ values, although with a higher frequency of gastrointestinal side effects during the first few weeks of treatment.

The greater glycaemic efficacy with liraglutide may derive from the different mechanism of action of GLP-1 receptor agonists and DPP-4 inhibitors. GLP-1 receptor agonists achieve greater (pharmacological) levels of GLP-1 activity, which, together with the extended half-life of liraglutide (13 h), results in effective, 24-h glucose control with once-daily dosing (10,11), as evidenced by the greater reductions in FPG. By contrast, DPP-4 inhibitors indirectly modulate endogenous GLP-1 and glucose-dependent insulinotropic polypeptide (GIP) concentrations by inhibiting the degradation of these peptide hormones by DPP-4. However, because individuals with T2D are resistant to GIP, endogenous GLP-1 may be insufficient for effective glycaemic control, which may partially explain the lower efficacy with DPP-4 inhibitors (12).

Greater weight reduction with liraglutide compared with sitagliptin can also be attributed to the mechanistic differences of GLP-1 analogues and DPP-4 inhibitors. Previous studies have shown that GLP-1 receptor agonists increase satiety, reduce food intake and promote weight loss (13,14), whereas DPP-4 inhibitors are generally weight-neutral (3).

Our efficacy findings over 52 weeks are supported by the results of other shorter head-to-head trials, where exenatide once weekly (DURATION-2) or taspoglutide (T-emerge 4) were compared with sitagliptin over 26 weeks (7,9). In these trials, HbA_1c_ was reduced by 1.5% and 1.3% with exenatide once weekly and taspoglutide, respectively, vs. 0.9% with sitagliptin. Thus, the overall efficacy results for sitagliptin in our trial are comparable, and for body weight change, even better, than the results of other trials that have generally shown DPP-4 inhibitors to be weight-neutral.

Trials investigating the ability of incretin-based therapies to provide early and sustained glycaemic management are important in light of recent findings that periods of poorly controlled hyperglycaemia increased future risk of diabetes-related death and complications (15–17). Our trial showed that liraglutide treatment initiation produced early and effective glycaemic control, as evidenced by HbA_1c_≤ 7% by week 12 and FPG ∼7.7–7.8 mmol/l (138.6–140.4 mg/dl) by week 4, and this control was generally maintained up to 52 weeks.

According to the ADA's latest Standards of Care, diabetes treatment needs to move beyond a glucocentric approach that focuses solely on controlling hyperglycaemia with minimal risk of hypoglycaemia (18). Optimised therapies should also address the comorbidities frequently associated with diabetes (i.e. obesity, hypertension and dyslipidaemia). Based on this multifactorial approach, we showed that, with liraglutide 1.8 mg, 50% of participants achieved HbA_1c_ < 7% with concomitant weight loss and minimal risk of hypoglycaemia, whereas only 19% of participants on sitagliptin were able to meet these criteria. While liraglutide did not significantly reduce systolic blood pressure compared with sitagliptin, the decrease in the 1.8 mg group was consistent with results of the LEAD-1 and LEAD-2 trials (−2.8 and −2.3 mmHg respectively) (11,19). The significant improvement in beta-cell function with liraglutide probably relates to reduced glucose toxicity because of improved glycaemic control and/or a direct effect on beta-cells, as has been shown in animal models with GLP-1 (20,21). The reduction in insulin resistance (HOMA-IR) from baseline with 1.8 mg liraglutide in our study (−1.36%) was similar to that in the 52-week LEAD-3 trial (−1.35%) (22), and may be related to body weight reduction. Overall, greater improvements were observed with 1.8 mg than 1.2 mg liraglutide after 52 weeks. The 1.2 mg dose did not decrease systolic blood pressure, did not significantly improve fasting C-peptide concentration or insulin resistance and enabled only ∼39% of patients to reach the abovementioned composite end-point.

Patient acceptance is a crucial element of treatment success. Although both doses of injectable liraglutide produced similar improvements in overall treatment satisfaction scores, only the improvement in the 1.8 mg group reached statistical significance compared with oral sitagliptin, similar to results at 26 weeks. The higher overall treatment satisfaction with liraglutide 1.8 mg vs. sitagliptin after 52 weeks may be attributed, in part, to the greater glycaemic and weight benefits of liraglutide, along with good tolerability and availability in a simple injection device. Greater treatment satisfaction with injectable GLP-1 therapy over oral DPP-4 therapy is consistent with findings from another trial (9). Patient satisfaction with treatment is important because it may offer a clinically valuable indication on treatment adherence (23), and relates to long-term outcomes.

In agreement with previous findings, both drugs were generally safe and well tolerated (24–26). Nausea was higher with liraglutide than with sitagliptin, most probably because of the greater (pharmacological) levels of GLP-1 activity. The decline in nausea incidence after the first 3 weeks is consistent with the transient nature of nausea upon initiation of GLP-1 therapy (24).

A small increase in heart rate (2–3 beats/min) occurred with liraglutide in this trial, similar to the 2–4 beats/min increases reported in previous studies (11,19,22,27,28). The clinical significance of this elevation is not clear, but a similar increase (∼2 beats/min) was reported with another GLP-1 receptor agonist, exenatide twice daily (29). Calcitonin and thyroid AEs were recorded because of potential concerns that originated in preclinical testing with GLP-1 receptor agonists. Consistent with previous clinical trials with liraglutide, calcitonin levels remained well below the upper limit of normal for both genders in this trial. In addition, clinical thyroid events were reported by few trial participants and the proportions of participants with these events were comparable between liraglutide (1.2 or 1.8 mg) and sitagliptin groups. No cases of thyroid cancer were reported.

Our study design had some limitations, including the absence of a placebo group to serve as a benchmark for some of the safety end-points and the lack of double-blinding. In addition, LOCF imputation of the 26-week data may have resulted in a conservative estimate of the 52-week effect of the study drugs. However, statistical analyses of the HbA_1c_ and body weight end-points performed using a completers analysis set produced similar results. LOCF is a commonly used method for imputing missing data and is transparent in the context of diabetes trials (30).

GLP-1 receptor agonists are given preference over DPP-4 inhibitors in dual- and triple-therapy intensification regimens after metformin by the AACE and by the ADA/European Association for the Study of Diabetes as a tier-2 therapy when hypoglycaemia and weight concerns are paramount (1,2). However, these guidelines do not mention liraglutide, as it was not approved at the time of publication. Furthermore, the current treatment algorithms are mostly based on the results of independent trials with either GLP-1 receptor agonists or DPP-4 inhibitors against other agents or placebo. Our trial offers the first 52-week, direct comparison of the overall clinical profiles of the two types of incretin therapy, and provides support for the use of GLP-1 receptor agonists as an efficacious alternative to DPP-4 inhibitors in treatment intensification algorithms.

In summary, 52 weeks of liraglutide treatment in combination with metformin provides sustained and superior glycaemic control and significant body weight reduction compared with sitagliptin in combination with metformin, while maintaining a comparable safety and tolerability profile, albeit with more gastrointestinal side effects initially, in participants with T2D.

## Supporting information

**Table S1.** Thyroid-related treatment-emergent adverse events by system organ class and preferred term.

**Table S2.** Thyroid-related treatment-emergent adverse events in the neoplasm system organ class.
