# Supplementary material for: One year of liraglutide treatment offers sustained and more effective glycaemic control and weight reduction compared with sitagliptin, both in combination with metformin, in patients with type 2 diabetes: a randomised, parallel-group, open-label trial
Source: Int J Clin Pract. 2011 Apr;65(4):397–407. doi: 10.1111/j.1742-1241.2011.02656.x (PMC3085127; doi:10.1111/j.1742-1241.2011.02656.x)
Supplement: Supplementary file 1 [file ijcp0065-0397-SD1.doc]

**Supplementary Table 1**. Thyroid-related treatment-emergent adverse events by system organ class and preferred term.

|  | **Liraglutide 1.2 mg**  **N (%) E** | **Liraglutide 1.8 mg**  **N (%) E** | **Sitagliptin**  **N (%) E** |
| --- | --- | --- | --- |
| Safety analysis set | 221 | 218 | 219 |
| Adverse event | 11 (5.0) 13 | 12 ( 5.5) 16 | 10 ( 4.6) 13 |
| Investigations | 9 ( 4.1) 9 | 10 ( 4.6) 13 | 6 ( 2.7) 7 |
| Blood calcitonin increased | 9 ( 4.1) 9 | 10 ( 4.6) 13 | 6 ( 2.7) 7 |
| Neoplasms benign, malignant and unspecified (including cysts and polyps) | 1 ( 0.5) 1 | 2 ( 0.9) 2 | 0 ( 0.0) 0 |
| Thyroid neoplasm | 1 ( 0.5) 1 | 2 ( 0.9) 2 | 0 ( 0.0) 0 |
| Endocrine disorders | 3 ( 1.4) 3 | 1 ( 0.5) 1 | 6 ( 2.7) 6 |
| Goitre | 2 ( 0.9) 2 | 1 ( 0.5) 1 | 4 ( 1.8) 4 |
| Hyperthyroidism | 0 ( 0.0) 0 | 0 ( 0.0) 0 | 1 ( 0.5) 1 |
| Thyroid cyst | 0 ( 0.0) 0 | 0 ( 0.0) 0 | 1 ( 0.5) 1 |
| Thyroid disorder | 1 ( 0.5) 1 | 0 ( 0.0) 0 | 0 ( 0.0) 0 |

Analysis was performed on the safety analysis set.

E, number of adverse events; N, number of subjects; %, proportion of subjects having adverse events.

A treatment-emergent adverse event is defined as an event occurring between first drug date and last drug date +7 days for subjects withdrawn before week 52 and between first drug date and week 52 for completers or starting before first drug date with increasing severity during the trial period.

**Supplementary Table 2**. Thyroid-related treatment-emergent adverse events in the neoplasm system organ class.

| System organ class | Preferred term | Treatment group | Onset (treatment days*) | Severity | Relation to trial drug? | Dose change | Outcome | Serious? |
| --- | --- | --- | --- | --- | --- | --- | --- | --- |
| Neoplasms benign, malignant and unspecified (including cysts and polyps) | Thyroid neoplasm/regressive thyroid gland (1 cyst, 3 calcified nodes) | Liraglutide 1.8 mg | 57 | Mild | Unlikely | No | Not recovered | No |
|  | Thyroid neoplasm/solitary nodule in thyroid gland | Liraglutide 1.8 mg | 184 | Mild | Possible | Product withdrawn | Not recovered | No |
|  | Thyroid neoplasm/thyroid nodules | Liraglutide 1.2 mg | 117 | Mild | Unlikely | No | Not recovered | No |

Analysis was performed on the safety analysis set. A treatment- emergent adverse event is defined as an event occurring between first drug date and last drug date +7 days for subjects withdrawn before week 52 and between first drug date and week 52 for completers or starting before first drug date with increasing severity during the trial period.

*Relative to first drug dose.
